# Minimally Invasive Resin-Bonded Inlay-Retained Cantilever Fixed Dental Prosthesis: A Clinical Report

**DOI:** 10.1155/2021/5578026

**Published:** 2021-05-28

**Authors:** Albandari Bin-Rubayan, Abdulaziz Samran, Ali Alqerban

**Affiliations:** ^1^Ministry of Health (SCFHS), Prosthodontics Program (Training Center: Department of Restorative and Prosthetic Dental Sciences, College of Dentistry, Dar Al Uloom University), Riyadh, Saudi Arabia; ^2^Department of Restorative and Prosthetic Dental Sciences, School of Dentistry, Dar Al-Uloom University, Riyadh, Saudi Arabia; ^3^Department of Prosthodontics, School of Dentistry, Ibb University, Yemen; ^4^Department of Preventive Dental Sciences, College of Dentistry, Prince Sattam Bin Abdulaziz University, Alkharj, Saudi Arabia; ^5^Department of Preventive Dental Science, Dar Al Uloom University, Riyadh, Saudi Arabia

## Abstract

This clinical report demonstrates the treatment of a healthy adult patient referred for prosthodontic treatment after orthodontic treatment with a resin-bonded inlay-retained cantilever fixed dental prosthesis (IRCFDP). The purpose of this report was to demonstrate the esthetic, functional, and conservative properties of a resin-bonded IRCFDP fabricated from monolithic zirconia which can be placed in posterior area in certain situations. Acceptable esthetics with a conservative and functional result were achieved by using a resin-bonded inlay-retained cantilever fixed dental prosthesis (IRCFDP). All laboratory and clinical procedures of this case report are described.

## 1. Introduction

Replacement of missing teeth area can be accomplished with resin-bonded fixed dental prostheses (FDPs), conventional fixed dental prostheses (FDPs), implant-supported FDPs, or removable dentures. Conventional FDPs typically require the removal of 50% to 70% of sound dental tissue [1, 2] which has been reported to lead to damage tooth vitality in 10% of the FDPs [3, 4]. For that reason, these options are indicated when the adjacent teeth are extensively restored or damaged. Tooth structure can be preserved with adhesively placed resin-bonded FDPs.

Metal resin-bonded fixed dental prostheses (RBFDPs) have been recommended as a conservative option to conventional FDPs [5–8]. However, when a 2-retainer design is used, debonding of one abutment often results in caries that is not perceived by the patient. Ceramic anterior RBFDPs were first described in the 1990s and have been used to replace posterior teeth in certain situations [9]. Ceramic cantilever RBFDPs were suggested by Kern et al. [10] since their clinical study of ceramic RBFDPs with two retainers exhibited high fracture rates within the first year after insertion. The cantilever design leads to reduced shear and tensile forces compared with splinting two abutments teeth with differential movement [11]. In addition, when cantilever RBFDPs debond, the patient will immediately notice [12]. In a report of 10-year follow-up study, the survival rate of cantilever ceramic RBFDPs was 98.2% [13], which was better than that of two-retainer RBFDPs [14]. A zirconia inlay-retained fixed dental prosthesis (IRFDP) design was suggested by Wolfart and Kern [15], and Bishtia et al. [16] tested a new design for inlay-retained cantilever fixed dental prostheses (IRCFDPs) in an in vitro study. This design represents a conservative solution which can be applied in some patients, such as those with existing restorations or caries and when an implant-supported prosthesis is contraindicated. The aim of this clinical report was to describe the replacement of a missing mandibular right first molar with a computer-aided design and computer-aided manufacture (CAD-CAM) resin-bonded IRCFDP made of zirconia ceramic.

## 2. Case Report

**Figure 1:**
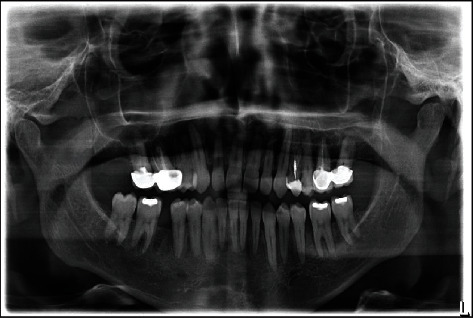
Pretreatment panoramic radiograph of initial patient presentation.

**Figure 2:**
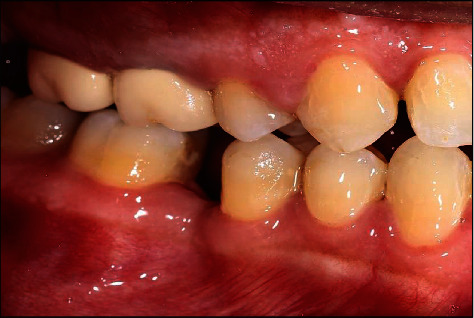
Pretreatment lateral view of mandibular arch.

**Figure 3:**
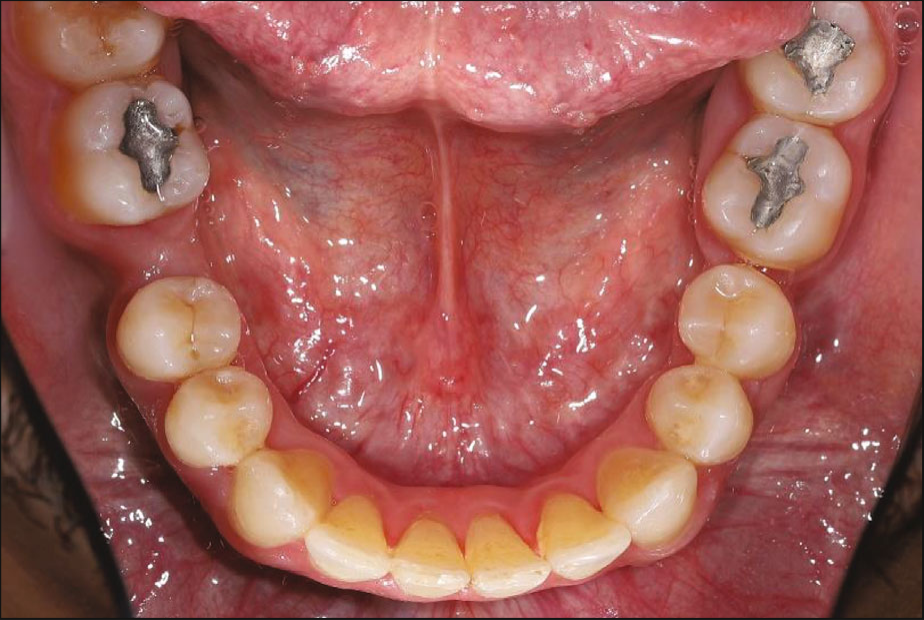
Pretreatment occlusal view of mandibular arch.

**Figure 4:**
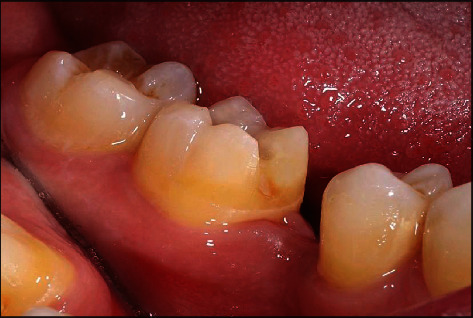
The mandibular right second molar after preparation.

**Figure 5:**
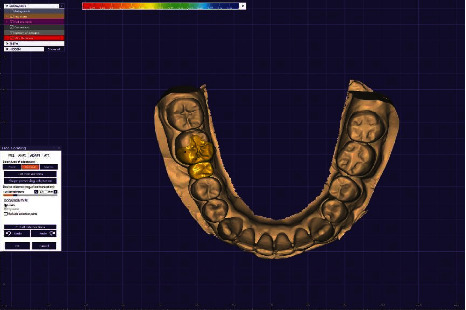
Computer-aided designed framework (occlusal view).

**Figure 6:**
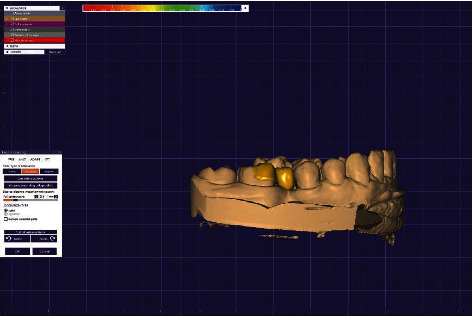
Computer-aided designed framework (buccal view).

A healthy 42-year-old man visited the Department of Prosthodontics of Dar Aluloom University seeking a replacement for his missing mandibular right first molar which had been extracted several years ago after unsuccessful endodontic treatment (Figure 1). His dental history also included orthodontic treatment for about two years to correct malocclusion (crowding in some areas and spacing in other areas because of his missing four first molars). The orthodontist referred him to the prosthodontic clinic to restore the narrow space in the mandibular right first molar area, which had not been closed by the orthodontic treatment. The clinical investigation revealed a narrow mandibular first molar space that contraindicated for implant placement (Figure 2). In addition, the mandibular right second molar had an occlusal amalgam restoration with occlusal recurrent caries (Figure 3). The majority of teeth were vital, and the oral hygiene was good. A periapical radiograph revealed a deep existing amalgam restoration in the mandibular right second molar, with no periapical abscesses or other significant findings. After making primary impressions for diagnostic casts and consultation with other specialists, a treatment plan was formulated to replace the missing tooth with a minimally invasive resin-bonded IRCFDP [17]. The patient was informed about the risk of the proposed treatment and its alternatives, including a conventional cantilever FDP and IRFDP. After rubber dam placement, the existing amalgam restoration on the second right molar and the caries were removed, providing a cavity for the inlay retainer, which followed the preparation principles for ceramic inlay restorations [18]. The inlay cavity was prepared without bevels with fine-grit diamond rotary instruments by removing sharp margins, smoothing the pulpal floor, and preparing two retainer-wings buccally and lingually. The enamel surface was reduced by approximately 0.5 mm to provide a 3 × 4 mm enamel area for bonding (Figure 4). The reduction was parallel to the path of insertion of the inlay retainer. After abutment preparation, polyvinyl siloxane impression material was used for the final impression (Take 1 Advanced; Kerr Corp) in a stock tray and poured with Type IV dental stone (Fujirock; GC Corp). The stone cast was scanned with a laboratory scanner. The resin-bonded IRCFDP was designed as an inlay retainer with buccal and lingual retainer wings and a second premolar pontic and milled from an A2 zirconia shade block (Cercon HT Full Contour Zirconia; Dentsply Sirona) (Figures 5 and 6).

**Figure 7:**
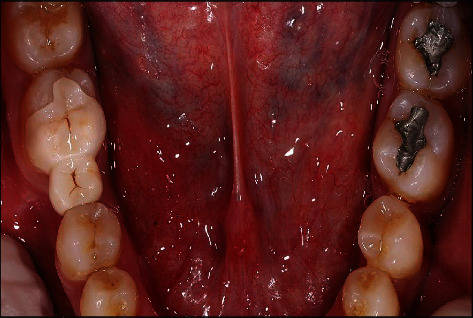
Evaluation of resin-bonded inlay-retained cantilever fixed dental prosthesis before cementation.

After sintering, the framework was seated on the cast after minor corrections, and then, the marginal fit and internal fit were checked intraorally using an explorer and a silicone indicator paste (Fit Checker, GC Corp). The silicone indicator paste exhibited a homogeneous and thin thickness which was accepted. For the inlay retainer, the minimum thickness was 3 mm, and for the buccal and lingual retainer wings, it was 0.7 mm. The dimensions for the proximal connector were approximately 4 mm in height and 4 mm in width. After try-in stage (Figure 7) and to remove the residues of the saliva and blood, the bonding surface of resin-bonded IRCFDP was cleaned using hot water steamer. Then, the bonding surface of the inlay retainer and the buccal and lingual retainer wings were airborne-particle abraded with 50 *μ*m Al_2_O_3_ for 10 seconds with 0.1 MPa pressure [19]. After that, the prosthesis was ultrasonically cleaned for 3 minutes in alcohol path to remove the abrasive residues. Consequently, the resin-bonded IRCFDP was stored in a special container while the preparation surfaces of the abutment tooth are cleaned and treated for the next cementation process.

**Figure 8:**
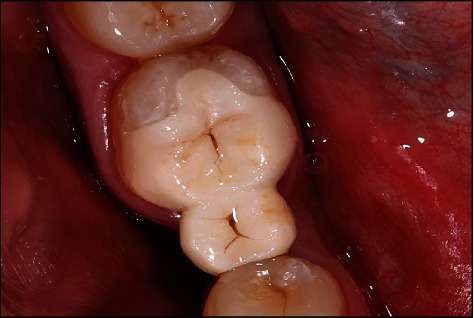
Occlusal view of resin-bonded inlay-retained cantilever fixed dental prosthesis immediately after insertion.

A rubber dam was applied during adhesive cementation, and the abutment tooth was cleaned with pumice. Then, the preparation surfaces of the abutment tooth were etched with a 37% phosphoric acid gel (Cica; Promedica) for 15-30 sec (according to the tooth structure; dentin or enamel). Then, the acid etch gel was sprayed off with water for 15 sec, and the tooth was thoroughly dried with air stream. After that, the enamel and dentin walls were conditioned with corresponding primer (Compobond LCM Primer; Promedica) which was mixed according to manufacturer's instructions and applied for 30 seconds before dispersing the excess using gentle oil-free air stream. Then, the adhesive material (Compobond LCM Adhesive; Promedica) was mixed according to manufacturer's instructions and applied for 15 seconds before removing the excess using gentle oil-free air stream and light cured for 15 sec. Finally, bonding surfaces of the prosthesis were primed with a ceramic primer (Aureocem DC Ceramic Primer; Promedica) using a microbrush. The primer was left for 60 s, and the excesses were removed with an oil-free air stream. After that, the cement (Aureocem DC Automix, Promedica) was distributed over the resin-bonded IRCFDP bonding surfaces, and the prosthesis was seated in place (Figure 8). Steady finger pressure was applied during the setting time. After cementation, the function and occlusion were checked using articulator papers.

## 3. Discussion

This clinical report describes replacing the narrow space of a missing mandibular right molar with an esthetic and conservative resin-bonded IRCFDP fabricated from monolithic zirconia. The esthetics of this zirconia resin-bonded IRCFDP were excellent, better than the metal-ceramic FDP. Although this technique has been recommended only as an interim technique of replacing of missing teeth, however, its conservative preparation, esthetic, and reported survival rate suggest that it may be considered as a definitive treatment choice in certain situations [6, 13, 20–22]. The framework of the current resin-bonded IRCFDP was made of monolithic zirconia material based on laboratory studies that reported higher fracture load for zirconia-based IRFDPs than those made from lithium disilicate ceramic [23–25]. This resin-bonded IRCFDP design was developed to overcome the high failure rate of previous IRFDP designs which might not be recommended for clinical indications [26–28]. The presence of wings in this design reduced stress on the inlay retainer caused by torsion forces applied nonaxially and to increase the enamel surface area for bonding. Similar retainer wings have been used in previous studies with cantilevered ceramic resin-bonded FDPs [20, 29].

Disadvantages of this design included the poor color match of the resin-bonded IRCFDP to the adjacent natural teeth because the prosthesis was made completely of monolithic zirconia (Figure 6). Additionally, the wings were somewhat bulky which added approximately 0.1 to 0.2 mm to the tooth contour. A suggested minimum thickness of 0.6 to 0.7 mm was used for the zirconia wings, and the tooth preparation was minimal in this area (approximately 0.5 mm) [17].

## 4. Conclusion

An esthetic and conservative approach to replacing the narrow space of a missing posterior molar with a resin-bonded IRCFDP fabricated from monolithic zirconia was described. After a conservative tooth preparation, a resin-bonded IRCFDP with buccal and lingual wings was fabricated from monolithic zirconia. The patient was so satisfied and pleased with the aesthetics and functional results. This treatment option helped maintain the abutment and the adjacent teeth. Clinical follow-up is important to determine the success rate of this type of prosthesis.
